# Differential Effects of Beta-Hydroxybutyrate Enantiomers on Induced Pluripotent Stem Derived Cardiac Myocyte Electrophysiology

**DOI:** 10.3390/biom12101500

**Published:** 2022-10-17

**Authors:** Matthew L. Klos, Wanqing Hou, Bernard Nsengimana, Shiwang Weng, Chuyun Yan, Suowen Xu, Eric Devaney, Shuxin Han

**Affiliations:** 1 Department of Hepatobiliary Surgery, Anhui Province Key Laboratory of Hepatopancreatobiliary Surgery, The First Affiliated Hospital of USTC, Division of Life Sciences and Medicine, University of Science and Technology of China, Hefei 230001, China; 2 Department of Endocrinology, The First Affiliated Hospital of USTC, Division of Life Sciences and Medicine, University of Science and Technology of China, Hefei 230001, China; 3 Pediatric Cardiac and Thoracic Surgery, University Hospitals Cleveland Medical Center, Cleveland, OH 44106, USA

**Keywords:** beta-hydroxybutyrate (βOHB), induced pluripotent stem cell-derived cardiac myocytes (iPS-CMs), enantiomer, racemic mixture, electrophysiology

## Abstract

Beta-hydroxybutyrate (βOHB), along with acetoacetate and acetone, are liver-produced ketone bodies that are increased after fasting or prolonged exercise as an alternative fuel source to glucose. βOHB, as the main circulating ketone body, is not only a G-protein coupled receptor ligand but also a histone deacetylases inhibitor, prompting the reexamination of its role in health and disease. In this study, we compared the effects of two commercial βOHB formulations an enantiomer R βOHB and a racemic mixture ±βOHB on induced pluripotent stem cell cardiac myocytes (iPS-CMs) electrophysiology. Cardiac myocytes were cultured in R βOHB or ±βOHB for at least ten days after lactate selection. Flouvolt or Fluo-4 was used to assay iPS-CMs electrophysiology. We found that while both formulations increased the optical potential amplitude, R βOHB prolonged the action potential duration but ±βOHB shortened the action potential duration. Moreover, ±βOHB increased the peak calcium transient but R βOHB reduced the peak calcium transient. Co-culturing with glucose or fatty acids did not ameliorate the effects, suggesting that βOHB was more than a fuel source. The effect of βOHB on iPS-CMs electrophysiology is most likely stereoselective, and care must be taken to evaluate the role of exogenous βOHB in health and disease.

## 1. Introduction

Energy consumption in mammals is derived largely from the consumption of three macronutrients; protein, carbohydrates, and fat [1]. During periods of reduced glucose availability caused by fasting, prolonged exercise, or a ketogenic diet, lipids are consumed for the hepatic production of ketone bodies [2].

The three ketone bodies are beta-hydroxybutyrate (βOHB), acetoacetate, and acetone [3]. βOHB and acetoacetate are produced in the liver from fatty acids, while acetone is produced by the spontaneous non-enzymatic decarboxylation of acetoacetate. While all three ketone bodies are present during a state of ketosis, βOHB is the main ketone body, constituting upwards of 70% of all circulating ketone bodies after a prolonged fasting [4]. Once released into the bloodstream, ketone bodies can freely diffuse across cell membranes [5]. In addition, ketone bodies can also enter the cells via monocarboxylate transporters (MCT1 and MCT2, also known as solute carrier 16A family members 1 and 7) [6]. After entering the mitochondria, βOHB and acetoacetate can be broken down into acetoacetyl-CoA and enter the citric acid cycle [7].

Traditionally, elevations in blood ketones have been viewed as pathogenic by the medical community because of their association with the diabetic ketoacidosis [8]. However, careful research discovered that ketone body production is an essential evolutionary conserved mechanism that restores normal cerebral function when carbohydrate stores are exhausted [9].

Compared to the brain, which normally relies on glucose as its main energy source, the heart in contrast is an omnivore, capable of utilizing amino acids, glucose, fatty acids, lactate, and ketone bodies to meet its energy demand [10]. While the healthy heart derives most of its energy demand from β-oxidation, it was recently discovered that the failing heart decreases its rates of β-oxidation and increases its rates of ketone body oxidation. At the time, it was unknown if this switch was physiological or pathogenic [11]. As the failing heart progressively loses its capacity to oxidize fatty acids and glucose, ketone bodies provide a potential alternative substrate, since ketone oxidation bypasses the complex dysregulation of the beta-oxidation pathway and pyruvate dehydrogenase complex in the failing heart. This suggests that ketone body metabolism has the potential to treat heart failure, a pathological state where the fetal genome is re-expressed [12]. We used concentrations of ketone bodies associated with short periods of fasting to investigate their physiological relevance.

A growing consensus is that the increase in ketone body oxidation is adaptive [13]. As for why a metabolite can act as a pharmaceutical, βOHB, in addition to being a more efficient fuel source, has also been found to be a histone deacetylase (HDAC) inhibitor, a ligand for the hydroxy-carboxylic acid HCA1/GPR81, HCA2/GPR109A, and HCA3/GPR109B, as well as a free fatty acid receptor, and an inhibitor of the NLRP3 inflammasome [14]. Stereospecific protein bindings and the accumulation of metabolic intermediaries caused by different rates of enantiomer metabolism are believed to be the reason for βOHBs pleiotropic effects [15].

βOHB is a chiral molecule existing as two enantiomers, R/d and S/l. The R/d enantiomer is the predominant enantiomer in circulation. In contrast, the S/l enantiomer is a short-lived intermediary created by the final round of fatty acid β-oxidation that is mostly converted into the R/d enantiomer [16]. Because S-enantiomer is “short-lived” and “mostly converted into the R enantiomer”, this can happen in the presence of R/S racemic mixture through an unknown molecular pathway, most likely involving the conversion of S- βOHB to acetyl-CoA and its subsequent conversion to R βOHB. It is currently unknown what role each enantiomer plays in cardiac development, health, and disease.

During normal human development, after birth, βOHB levels rapidly rise until they gradually decrease after the first two to three days of lactation [17]. During this time, there starts to be a profound switch in cardiac metabolism, where the heart goes from primarily consuming glucose to fatty-acids [18]. Associated with this metabolic switch are changes in the cardiac epigenome and cardiac excitation-contraction coupling [19]. As the heart develops, both the myocyte action potential amplitude and duration increase [20]. Likewise, calcium flux changes as well [20]. It is currently unknown how or if βOHB alters these parameters.

Therefore, in this study, we decided to test the effects of two commercially available different βOHB formulations, a pure R enantiomer and a stereoisomer mix on induced human pluripotent stem cell-derived cardiac myocytes (iPS-CMs) electrophysiology. In addition to being a human cell system, iPS-CMs are phenotypically identical to neonatal cardiac myocytes, making them the ideal reductionist model to study human cardiac development. We tested the hypothesis that culturing iPS-CMs with βOHB will metabolically mature iPS-CMs electrophysiology.

## 2. Materials and Methods

### 2.1. iPS-CMs Generation and Purification

iPS-CMs were generated similarly to the protocol described by Burridge et al. [21]. iPS cells were grown on Matrigel-coated plates in Essential 8 media until they were approximately 85% confluent. Then the media was changed to RPMI + B27-insulin + 6 µM CHIR99021 (ThermoFisher, Selleck Chemicals LLC, Waltham, MA, USA), a GSK3B inhibitor, and conical Wnt signaling pathway activator. After 48 h, the media was then changed to RPMI + B27-insulin. After 24 h, the media was then changed to RPMI + B27-insulin + 2 µM Wnt-C59 (ThermoFisher, Selleck Chemicals LLC), a Wnt signaling pathway inhibitor. After 48 h, the media was changed to RPMI + B27, which was changed every other day until 10 days after the start of differentiation. iPS-CMs were then metabolically selected by being cultured in RPMI media without glucose + B27 and 5 mM Lactate (ThermoFisher, MilliporeSigma, Burlington, MA, USA) for 10 days, and the media was changed every other day [22]. After metabolic selection, which results in cultures greater than 90% cTNT positive cells determined by flow cytometry, cells were dissociated with TrypLE Express (ThermoFisher) and plated onto Matrigel-coated coverslips (Corning, Corning, NY, USA) at a density of 125,000 cells per monolayer.

### 2.2. Immunofluorescence

iPS-CMs were fixed in formalin for 10 min before being washed 3X with PBS. Cells were then permeabilized with PBS containing 0.25% Triton X-100. After permeabilization, cells were incubated in a blocking buffer (Santa Cruz Biotechnology, Dallas, TX, USA) for 1 h before being incubated with anti-troponin I (MilliporeSigma), Anti-Sarcomeric Myosin (Developmental Studies Hybridoma Bank, Iowa City, IA, USA), and anti-alpha actinin (MilliporeSigma) overnight according to manufacturer’s instructions. Coverslips were then washed 3X in PBS before being incubated with Donkey anti-Mouse Alexa Fluor 488 (Thermo Fisher) for an hour before being washed 3X in PBS and stained with Dapi (MilliporeSigma). Coverslips were then imaged using an EVOS cell imaging system.

### 2.3. βOHB Media

After being metabolically selected, iPS-CMs were cultured in one of the following 6 media for a minimum of 10 days. See Table 1 for a full list of stock numbers. Control media (RPMI + B27), FA media (RPMI no glucose + B27 + 100 μM oleic acid + 50 μM palmitic acid + 11.1 mM galactose + BSA) βOHB media (RPMI no glucose + B27 + 5.5 mM βOHB), FA + Glucose media (RPMI + B27 + 100 μM oleic acid + 50 μM palmitic acid + 11.1 mM galactose + BSA), βOHB + Glucose media (RPMI + B27 + 5.5 mM βOHB), or βOHB + FA + Glucose media, (RPMI + B27 + 100 μM oleic acid + 50 μM palmitic acid + 5.5 mM βOHB + BSA).

### 2.4. Voltage and Calcium Recordings

Voltage and calcium recordings were made using an IonOptix recording system, as described previously by Han et al. [23]. For voltage recordings, iPS-CMs were loaded with FluoVolt in Tyrode’s solution (148 mM NaCl, 0.4 mM NaH_2_PO_4_, 1 mM MgCl_2_, 5.5 mM Glucose, 5.4 mM KCl, 1.8 mM CaCl_2_, 15 mM HEPES) with a 1:1000 dilution of the FluoVolt dye (component A) and 1:100 of the PowerLoad (component B) for 25 min at room temperature before imaging. For calcium recordings, Fluo-4 was loaded at a concentration of 10 µM dissolved in DMSO and 10% pluronic F-127 in Tyrode’s solution for 20 min at 37 degrees, followed by a 20-min washout in Tyrode’s solution at room temperature.

After dye loading, iPS-CMs were placed on an inverted microscope, perfused with Tyrode’s solution, and paced at 3 Hz using field stimulation. A 40X objective was used to image iPS-CMs. Because both FluoVolt and Fluo-4 emission/excitation spectrum work with the standard FITC settings, dye-loaded cells were excited using the IonOptix’s HyperSwitch Light Source (75W xenon-arc bulb) and the DP-Fluo Package (Excitation/Emission). To avoid phototoxicity, neutral density filters were used to limit the intensity of the light, and recordings were limited to 5 s in duration. iPS-CMs fluorescence emission wavelengths were recorded using a photomultiplier tube.

### 2.5. Data Analysis

Voltage and Calcium transients were analyzed using IonOptix’s IonWizard software. Only cells that followed 1:1 pacing were included. For voltage recordings, the upstroke velocity, peak amplitude, and action potential durations of 30, 50, and 90 percent (APD 30, APD 50, and APD 90) were measured. For calcium recordings, peak calcium, halfwidth, and reuptake times were calculated.

### 2.6. Statistics

Data were presented as the mean ± SE. Unpaired t-tests with a Bonferroni correction were used to test for statistical significance. A *p*-value < 0.008 was considered statistically significant.

## 3. Results

### 3.1. βOHB Alters iPS-CMs Optical Action Potentials

**Figure 1:**
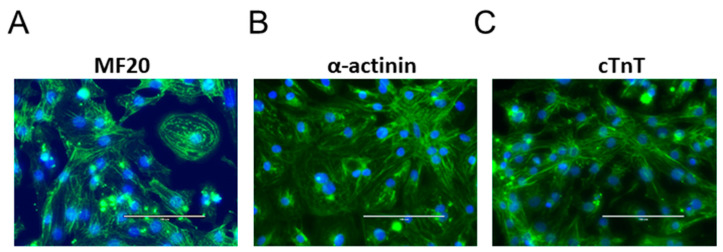
40X images of iPS-CMs stained for the cardiac markers α-actinin, myosin, and cardiac troponin T. (**A**) myosin, (**B**) α-actinin, and (**C**) cTnT are all positive.

Figure 1A–C shows images of iPS-CMs acquired using a 40X objective on an EVOS cell imaging system. After lactate selection, almost all the cultures were positive for cardiac α-actinin, cTnT, and myosin, indicating robust differentiation and metabolic purification.

Next, we cultured iPS-CMs in 6 different conditions. The base media used was RPMI 1640 with/without glucose. The control media refers to RPMI 1640 with glucose (11.1 mM). This also served as the base media for glucose-containing fatty acid and βOHB media. While 11.1 mM is considered to be prediabetic, it is an almost universally accepted iPS-CMs culture basal media [21]. RPMI media without glucose was the base media for the fatty acid and βOHB media.

To test the effects of βOHB on iPS-CMs electrophysiology, we cultured cells without glucose but with 5.5 mM ±βOHB or R βOHB as its primary carbon source. This amount was chosen since it approximates the steady state level of βOHB during a prolonged fasting [3]. Because fatty acids have been shown to metabolically mature iPS-CMs physiology, we included this group as an added control [24]. Further, because we previously demonstrated the addition of glucose alters ±βOHB’s effect on excitation-contraction coupling, we also cultured iPS-CMs in media containing βOHB, glucose, and fatty acids [25].

**Figure 2:**
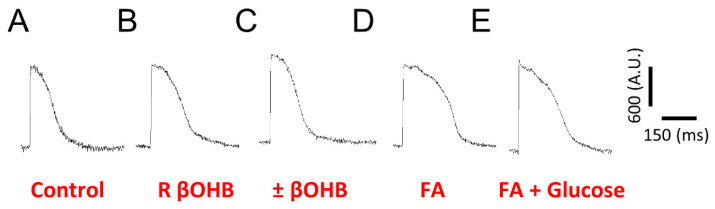
Ensembled averaged representative FluoVolt optical action potentials (AP) recorded from iPS-CMs cultured in either (**A**) control media, (**B**) R βOHB, (**C**) ±βOHB, (**D**) fatty acid media, or (**E**) fatty acid + glucose media.

As shown in Figure 2A–C, different media resulted in different action potential morphologies. Compared to the control conditions, the addition of fatty acids or βOHB increased the action potential amplitude.

**Figure 3:**
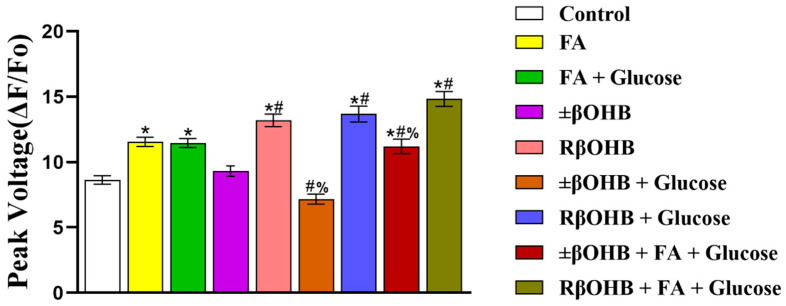
Quantification of FluoVolt optical AP amplitudes recorded from iPS-CMs cultured in various media. Peak voltage under control media, fatty acid media, fatty acid + glucose media, ±βOHB, R βOHB, ±βOHB + glucose media, R βOHB + glucose, ±βOHB + FA + glucose media, or R βOHB + FA + glucose media treatment. * *p* < 0.008 vs. control. **#** *p* < 0.008 vs. ±βOHB. **%** *p* < 0.008 vs. R βOHB.

However, in Figure 3, the addition of glucose to the R βOHB did not blunt the increase in the action potential amplitude. In addition, in the presence of glucose also makes no difference in R βOHB in the upstroke velocity.

**Figure 4:**
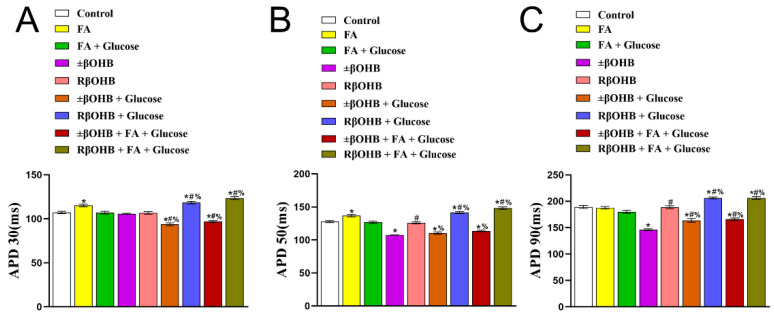
Quantification of FluoVolt optical AP durations (APD) recorded from iPS-CMs cultured in various media. The time it takes for an action potential to complete 30% repolarization (APD30) (**A**), the time it takes for an AP to complete 50% repolarization (APD50) (**B**), the time it takes for an AP to complete 90% repolarization (APD90) (**C**). * *p* < 0.008 vs. control. **#** *p* < 0.008 vs. ±βOHB. **%** *p* < 0.008 vs. R βOHB.

We next quantified the changes in the action potential duration. Compared to the control conditions, fatty acids prolonged the APD 30 and APD 50 (Figure 4A,B). Interestingly, the addition of glucose to the fatty acids media resulted in a slight decrease in APD 90 (Figure 4C). Added R βOHB unchanged the APD 30, APD 50, and APD 90, but the addition of glucose and/or fatty acids prolonged the APD 30, APD50, and APD 90 (Figure 4A–C). Conversely, the addition of ±βOHB resulted in a decrease in the APD 30, APD50, and APD90 (Figure 4A–C). However, the addition of glucose and/or fatty acids weakened the decrease in APD 30, APD 50, and APD 90 (Figure 4).

### 3.2. βOHB Alters iPS-CMs Calcium Transients

**Figure 5:**
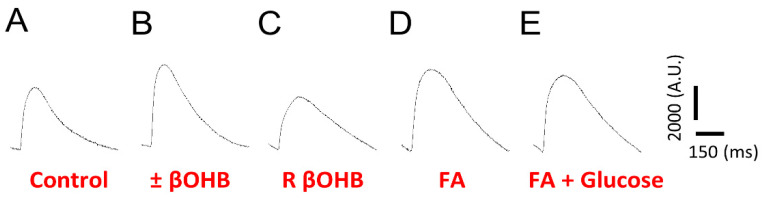
Ensembled averaged representative Fluo4 calcium transients recorded from cultured in either (**A**) control media, (**B**) ±βOHB, (**C**) R βOHB, (**D**) fatty acid media, or (**E**) fatty acid + glucose media.

**Figure 6:**
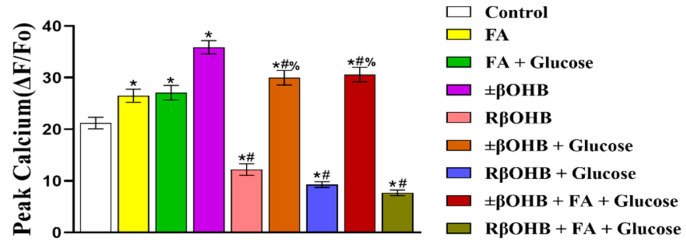
Quantification of Fluo4 calcium transient (Ca^2+^) amplitudes recorded from iPS-CMs cultured in various media. Peak calcium under control media, fatty acid media, fatty acid + glucose media, ±βOHB, R βOHB, ±βOHB + glucose media, R βOHB + glucose, ±βOHB + FA + glucose media, or R βOHB + FA + glucose media treatment. * *p* < 0.008 vs. control. **#** *p* < 0.008 vs. ±βOHB. **%** *p* < 0.008 vs. R βOHB.

After examining the effects of βOHB on the optical action potentials, we then decided to examine its effects on the calcium transient. Similar to the optical action potentials, culturing iPS-CMs in different media results in different calcium transient waveforms. Compared to optical action potentials, calcium transients have a slower upstroke time and longer decay time. While both the fatty acid media and ±βOHB increased the calcium transient amplitude, the R βOHB decreased it (Figure 5A–E). This effect was observed even when glucose and/or fatty acids were added (Figure 6).

**Figure 7:**
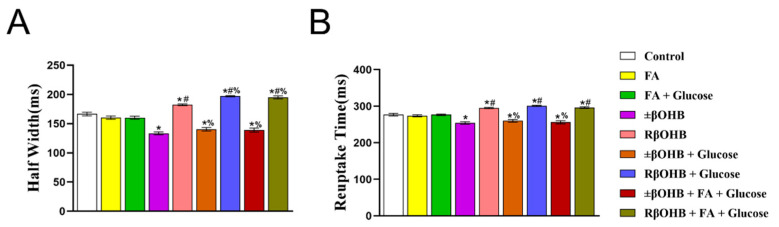
Quantification of Fluo4 Ca2+ half-widths and reuptake times recorded from iPS-CMs cultured in various media. (**A**) half-width: the time between 50% time to peak and 50% time to baseline, (**B**) reuptake time: 90% return to baseline. * *p* < 0.008 vs. control. # *p* < 0.008 vs. ±βOHB. % *p* < 0.008 vs. R βOHB.

After quantifying the amplitudes, we next examined the effects of βOHB on the calcium transient kinetics. Compared to the control condition, ±βOHB reduced both the half-width, defined as the time between 50% time to peak and 50% time to baseline. ±βOHB also shortened the reuptake time, defined as a 90% return to baseline. This was observed regardless of whether glucose and/or fatty acids were present in the media. Conversely, R βOHB had the opposite effect, and increased both the half-width and the reuptake time, regardless of the cultural situation (Figure 7A,B).

## 4. Discussion

**Figure 8:**
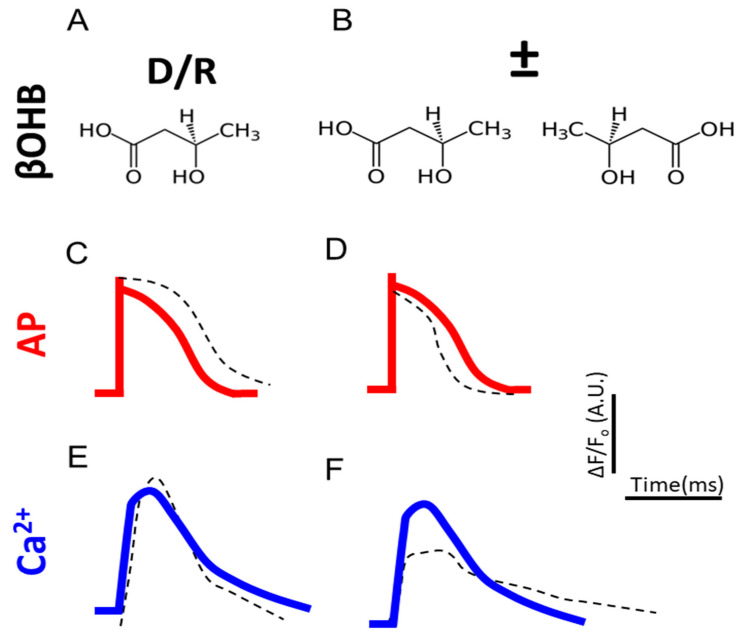
Summary of experimental findings. (**A**,**B**) Structural formulas for R βOHB and ±βOHB. (**C**,**D**) Quantification of FluoVolt optical AP amplitudes and durations of R βOHB and ±βOHB. (**E**,**F**) Quantification of Fluo4 Ca^2+^ amplitudes and kinetics of R βOHB and ±βOHB. The red and blue lines represent controls.

The main findings of this study are that the R enantiomer of βOHB alters iPS-CMs electrophysiology differently in contrast to a racemic mix (Figure 8). While both βOHB mixtures increased the amplitude of the voltage transient, the racemic mix shortened the APD 30, APD 50, and APD 90, but the R enantiomer prolonged the APD. Although the racemic mix increased the peak calcium transient, the R enantiomer reduced it. Likewise, while the racemic mix shortened both the calcium transient half-width and reuptake time, the R enantiomer prolonged both. The differences in action potential parameters or calcium transients hinted at changes in calcium channels.

Normally, circulating levels of βOHB are in the micromolar range. However, after periods of fasting or prolonged exercise βOHB can rise to 7 mM, or in the case of diabetic ketoacidosis, 20 Mm [26]. While the association with diabetic ketoacidosis has given ketosis a morbid undertone, it is now known that ketone body utilization is an evolutionarily conserved survival mechanism. Further, both βOHB and acetoacetate can act as signaling molecules in addition to being a more efficient fuel source, utilizing fewer molecules of oxygen than other substrates in the production of ATP [27]. As a result, there is a growing consensus that ketosis might be beneficial for the treatment of a variety of different diseases.

For over a century, it has been known that nutritional ketosis could successfully reduce and even eliminate seizures in patients with drug-refractory epilepsy [28]. A ketogenic diet has also been shown to be beneficial in treating a variety of other neurological conditions other than epilepsy such as Parkinson’s disease, multiple sclerosis, depression, aging, cancer, and heart disease [29,30]. Nevertheless, as opponents of a ketogenic diet correctly point out, plant-based diets are beneficial in treating the same diseases [31]. Arguably, this is because other metabolites have pleiotropic effects as well. Consequently, multiple diets may result in beneficial changes to human physiology via an unknown convergent molecular pathway.

Correia et al. discovered that culturing iPS-CMs with fatty acids as their primary carbon source increased the action potential amplitude, a reduction in the calcium transient amplitude, and alterations in the decay of the fluorescence signal for both the voltage and calcium signal [32]. Associated with this were increases in the expression of numerous ion channel transcripts, including ATP2A2, KCNJ2, and RYR2. Further investigation by Hu et al. not only found the same, but was able to demonstrate that it was because of a decrease in HIF1α levels [33].

In this study, while we found that fatty acids increased the AP amplitude and prolonged the APD 30 and APD 50, they did not prolong the APD 90, which contradicts their previously published results [34]. Likewise, while we also found that culturing iPS-CMs in fatty acids increased the peak calcium transient, we did not find differences in the half-width or reuptake time. While genomic analysis of alterations in ion channel transcription was beyond the scope of this study, these discrepancies are most likely the result of different recording conditions.

We recorded voltage and calcium transients from iPS-CMs paced at 3 Hz, the upper limit of the fetal resting heart rate, while Hu and Correia recorded their transients in iPS-CMs paced at 1 Hz, the extreme low end of the fetal resting heart rate. Excitation-contraction kinetics are rate dependent, and therefore is the most probable explanation for the discrepancies. Regardless, it highlights a limitation with using iPS-CMs.

Currently, there are no consensus on which protocol should be used to generate iPS cells, which iPS cell lines should be used for research, how many iPS cell lines should be used for a given study, which iPS base media should be used, which differentiation protocols should be used to generate iPS-CMs, which supplements should be included in the iPS-CMs media, how many independent differentiations should be used in a study, and at which time point after differentiation to use iPS-CMs. While consensus statements are emerging on how to use this technology, they are currently recommendations and not obligatory.

Irrespective, the metabolic selection of cardiac myocytes appears to be highly reproducible. Tohyama et al. study demonstrating that cultures of 99% cTnT positive cells can be obtained by replacing glucose with lactate has been cited 494 times according to Scopus at the time of this publication [22]. Likewise, it appears metabolic maturation of iPS-CMs by fatty acids is also highly reproducible [35]. Whether the effects of βOHB on iPS-CMs physiology are reproducible needs further investigation. Nevertheless, in this preliminary study, we demonstrate that two different formulations of βOHB have opposing effects on iPS-CMs electrophysiology. This is important for three reasons.

First, it suggests that the chirality of βOHB is important for its biological function in the heart. Second, βOHB may play a role in cardiac development. Third, it highlights the fact that the racemic formulation of βOHB matters. Currently, there is no consensus on which formulation of βOHB is utilized in scientific studies. Moreover, many studies do not use commercially available formulations but custom-synthesize their βOHB [36].

Therefore, we conclude that the βOHB enantiomer may be key for understanding the role of βOHB in cardiac development and understanding possible discrepancies in the reproducibility of studies involving nutritional ketosis achieved via exogenous supplementation versus a ketogenic diet. Studying cell metabolism under different culture conditions is what we are going to explore next. Understanding this information will be more conducive to the use of ketone bodies to fully utilize their physiological functions.

## Figures and Tables

**Table 1:** List of special reagents used for this study.

Component	Company	Number
Anti-Sarcomeric Myosin	Developmental Studies Hybridoma Bank	MF20
(R)-(−)-3-Hydroxybutyric acid sodium salt	MilliporeSigma	298360-1G
(±)-Sodium 3-hydroxybutyrate	MilliporeSigma	54965-10G-F
Sodium L-lactate	MilliporeSigma	L7022-5G
Oleic acid	MilliporeSigma	O1383-1G
Palmitic acid	MilliporeSigma	P0500-10G
D-(+)-Galactose	MilliporeSigma	G5388-100G
Anti-α-Actinin (Sarcomeric)	MilliporeSigma	A7732
Anti-Troponin T (Cardiac Muscle)	MilliporeSigma	MABT368
CHIR-99021 5 mg	Selleck Chemicals LLC	S1263
Thiazovivin 5 mg	Selleck Chemicals LLC	S1459
Wnt-C59 (C59) 5 mg	Selleck Chemicals LLC	S7037
Essential 8 Medium	Thermo Fisher Scientific	A1517001
Growth Factor Reduced Matrigel, 10 ml	Thermo Fisher Scientific	CB 40230
B 27 Supplement	Thermo Fisher Scientific	17 504 044
Gibco RPMI 1640 Medium	Thermo Fisher Scientific	11 875 093
Gibco RPMI 1640 Medium, no glucose	Thermo Fisher Scientific	11 879 020
Fluo-4, AM	Thermo Fisher Scientific	F14201
FluoVolt	Thermo Fisher Scientific	F10488
Donkey anti-Mouse IgG Alexa Fluor 488	Thermo Fisher Scientific	A-21202

## Data Availability

Not applicable.
